# Abundance, Diversity and Phenology of Mosquito Larvae in a Highly Anthropized Wetland: Health and Management Implications of Gándaras de Budiño (NW Spain)

**DOI:** 10.1002/ece3.71672

**Published:** 2025-06-26

**Authors:** Yasmina Martínez‐Barciela, Alejandro Polina, Josefina Garrido

**Affiliations:** ^1^ Departamento de Ecoloxía e Bioloxía Animal Universidade de Vigo Vigo Spain

**Keywords:** Culicidae, ecology, epidemiological risk, polluted waters, vector, West Nile virus

## Abstract

Because of their role as vectors of diseases, the presence of mosquitoes in wetlands often discourages their protection and conservation. The Gándaras de Budiño (Galicia, northwestern Spain) is a natural wetland highly damaged by the activity of a large industrial estate. The gathering in the same area of human hosts and migratory birds susceptible to carrying diseases such as West Nile virus (WNV) makes it necessary to determine the epidemiological risk of the wetland by studying the abundance, diversity and phenology of its culicid fauna. The field research was conducted in 11 water bodies sampled approximately every 15 days between January and December 2022 using the standardized dipping technique to capture mosquito larvae. A total of 766 specimens belonging to 11 species of the genus *Culex* (57%), *Culiseta* (30%) and *Anopheles* (13%) were identified, though the isolated observation of adult specimens of *Coquillettidia* and *Aedes* lead to the determination of eight functional groups. Mosquito abundance and diversity were significantly higher in temporary water bodies and spring season (with a median of 1–2 species more). Generalized linear mixed models (GLMM) revealed that both larval abundance and species richness were positively related with maximum ambient temperature and negatively with the mean relative humidity of the 15 days prior to the sampling date. Although almost all functional groups contain some mosquito capable of transmitting diseases, only *Cx. pipiens* s.l. (*N* = 287, 37.5%) and *Cx. torrentium* (*N* = 34, 4.4%) represent an emerging risk in the wetland due to their high competence to transmit WNV. Temporary and polluted waters were identified as the main breeding sites of these species, while spring and summer were the period of their major larval activity. The protection and proper management of natural wetlands not only favor the conservation of biodiversity, but is consistent with the prevention and control of mosquito‐borne diseases.

## Graphical abstract

The natural and anthropized wetland of Gándaras de Budiño (Galicia, Spain) hosts a great number of mosquito species. *Culex pipiens* s.l. and *Culex torrentium* pose an emerging risk for West Nile virus transmission. The temporary and polluted waters, as well as the summer and spring seasons, favor the larval proliferation of these vectors.

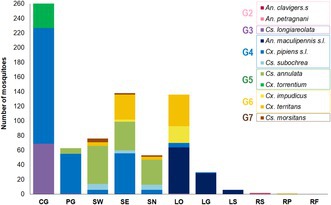

## RESUMEN

Debido a su rol como vectores de enfermedades, la presencia de mosquitos en los humedales a menudo desalienta su protección y conservación. Las Gándaras de Budiño (Galicia, noroeste de España) es un humedal natural enormemente dañado por la actividad de un gran polígono industrial. La presencia de hospedadores humanos y aves migratorias susceptibles de portar enfermedades como el Virus del Nilo Occidental (VNO) en la zona, hace necesario evaluar su riesgo epidemiológico mediante el estudio de la abundancia, diversidad y fenología de su Culicidofauna. El área de estudio se desarrolló en 11 cuerpos de agua muestreados aproximadamente cada 15 días entre enero y diciembre de 2022 mediante la técnica estandarizada de *dipping* para la captura de larvas de mosquitos. Se identificaron un total de 766 ejemplares pertenecientes a 11 especies del género *Culex* (57%), *Culiseta* (30%) y *Anopheles* (13%), aunque la observación aislada de ejemplares adultos de *Coquillettidia* y *Aedes* llevó a la determinación de ocho grupos funcionales. La abundancia y diversidad de mosquitos fue significativamente mayor en cuerpos de agua temporales y durante la primavera (entre 1 y 2 especies más). Los modelos lineales generalizados (GLMM) revelaron que tanto la abundancia larvaria como la riqueza de especies estuvieron positivamente relacionados con la temperatura máxima ambiental y negativamente con la humedad relativa de los 15 días previos a la fecha de muestreo. Aunque la mayor parte de los grupos funcionales contienen alguna especie capaz de transmitir enfermedades, sólo *Cx. pipiens* s.l. (*N* = 287, 37.5%) y *Cx. torrentium* (*N* = 34, 4.4%) representan un riesgo emergente en el humedal debido a su competencia para transmitir el VNO. Las aguas temporales y contaminadas fueron identificadas como los criaderos principales de estas especies, mientras que la primavera y el verano fueron los periodos de mayor actividad larvaria. La protección y el adecuado manejo de los humedales naturales no sólo favorece la conservación de la biodiversidad, sino que refuerza las medidas de prevención y control frente a las enfermedades transmitidas por mosquitos.

## 1 Introduction

Wetlands are valuable environments that harbor great biological diversity and provide important ecological, economic and social services (water storage, flood control, carbon sequestration, etc.) (Rey et al. 2012). Since the immature stages of mosquitoes are strictly aquatic, their populations are strongly linked to wetlands (Dale and Knight 2008). Despite their important role in the ecology of these environments (primary productivity and nutrient cycling, food source, pollination, etc.) (Mazzacano and Black 2013), mosquito presence often discourages the conservation and maintenance of wetlands due to their potential for annoyance and their role as vectors of human and animal diseases (Schäfer et al. 2004). In fact, many of these areas have historically been destroyed in Europe in an attempt to eradicate malaria, transmitted by *Anopheles* mosquitoes (Sousa et al. 2009). However, the mosquito‐borne disease that is currently most concerning for its reappearance in several European countries is West Nile virus (WNV), transmitted by *Culex* species and linked to the migration of wild birds from SubSaharan Africa to temperate regions of Europe (Malkinson and Banet 2002; Watts et al. 2021). Over the last two decades, WNV has become endemic in southern Spain with a noteworthy increase in seroprevalence in birds during the last years (Magallanes et al. 2024). Considering the effects of climate change, which increases the frequency and intensity of droughts, it is expected that bird populations will be affected by dispersing to other wetlands with better hydrological conditions (Almaraz and Green 2024; Magallanes et al. 2024). The Autonomous Community of Galicia is a highly humid region in northwestern Spain that contains the largest number of registered wetlands in the Iberian Peninsula (Gómez‐Orellana et al. 2014), making it an interesting migration and resting area for birds (Álvarez and Ramón 2009).

The present study of mosquito populations in a Galician wetland—Gándaras de Budiño—responds to the need of raising awareness, assessing health risks, and promoting the good management of these wet areas of great cultural and natural value, which are often exploited and neglected, resulting in avoidable negative health implications for humans and animals. Due to its topography and climate, this wetland was historically an extensive humid area with hunting interest (Silva‐Pando et al. 1987) and important human settlements (Lombera‐Hermida et al. 2018). In the mid‐20th century, this area began to be dismantled and fragmented by different communication paths which promote transit between southern Galicia and northern Portugal (the Vigo‐Monforte railroad, the N‐550 road, and the A‐55 highway). In the 1960s, two industrial estates were built, which destroyed a large part of the wetland and contaminated its ecosystems with different types of pollutants (Silva‐Pando et al. 1987). At the beginning of the 21st century, this area was included in the Natura 2000 Network as a Site of Community Interest (DOCE 2004) and, more recently, as a Special Area of Conservation (DOG 2014) due to its great richness of flora and fauna, pointing out the presence of certain species of conservationist interest such as *Cerambyx cerdo* Linnaeus, 1758, *Chioglossa lusitanica* Bocage, 1864, *Emys orbicularis* (Linnaeus, 1758), *Galemys pyrenaicus* Geoffroy, 1811, and *Lutra lutra* Linnaeus, 1758. Even so, the wetland remains mistreated and without adequate management to this day, showing that there is still no real awareness of the natural importance of this space. More studies are needed to reflect the diversity of the different organisms present in this area, as has already been documented for Fungi (Requejo and Castro 2017) and Coleoptera (Pérez‐Bilbao and Garrido 2009). However, the study of mosquitoes has been neglected and limited to isolated observations made between 2005 and 2008 based on an unadapted methodology for their capture (Martínez‐Barciela et al. 2021, 2023). Since not all mosquito species are disease vectors and do not produce the same nuisance (Mazzacano and Black 2013), knowing the diversity, abundance, distribution, and seasonal dynamics of the different species present in a wetland is essential to assess health risks (Rey et al. 2012). Therefore, mosquito surveillance in wetlands is crucial to implement the most appropriate vector management and control measures (Dale and Knight 2008; Dwork et al. 2022).

## 2 Materials and Methods

### 2.1 Study Area

**FIGURE 1:**
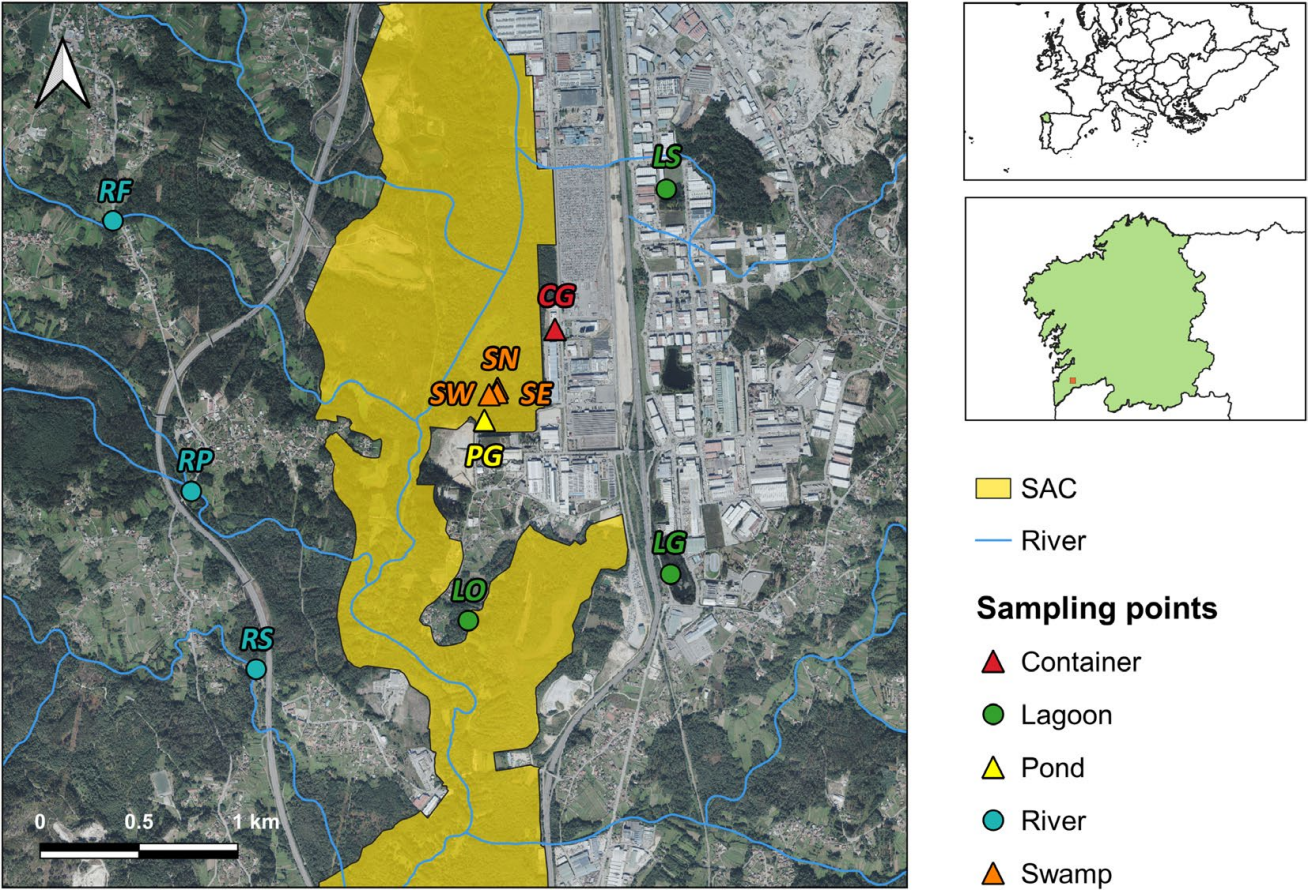
Sampling points in the study area on satellital map (Gándaras de Budiño, Galicia, Spain). Codes refer to the sampling points' names indicated in Table 1. SAC, Special Area of Conservation (DOG 2014).

The Gándaras de Budiño is a wetland that occupies an area of more than 700 ha between the municipalities of O Porriño, Tui, and Salceda de Caselas, in the southwest of the Pontevedra province (Galicia, northwestern Spain) (Figure 1). Although located in the Atlantic biogeographic region of Europe, this area is classified within the warm‐summer Mediterranean climate (Csb) according to Köppen‐Geiger climate classification, characterized by cold or mild winters, dry and cool summers, and seasonal rainfall (Köppen 1900). The seasonal rising of the Louro River (a tributary of the Miño River, the main fluvial course of the Galician hydrographic network) favors the flooding of this valley, leading to the formation of stagnant waters (ponds, swamps and lagoons) on a predominantly clayey soil (Silva‐Pando et al. 1987). However, the great anthropic pressure on the wetland in the last decades, together with climate change effects, has drastically reduced its water surface to less than 100 ha (Requejo and Castro 2017).

**FIGURE 2:**
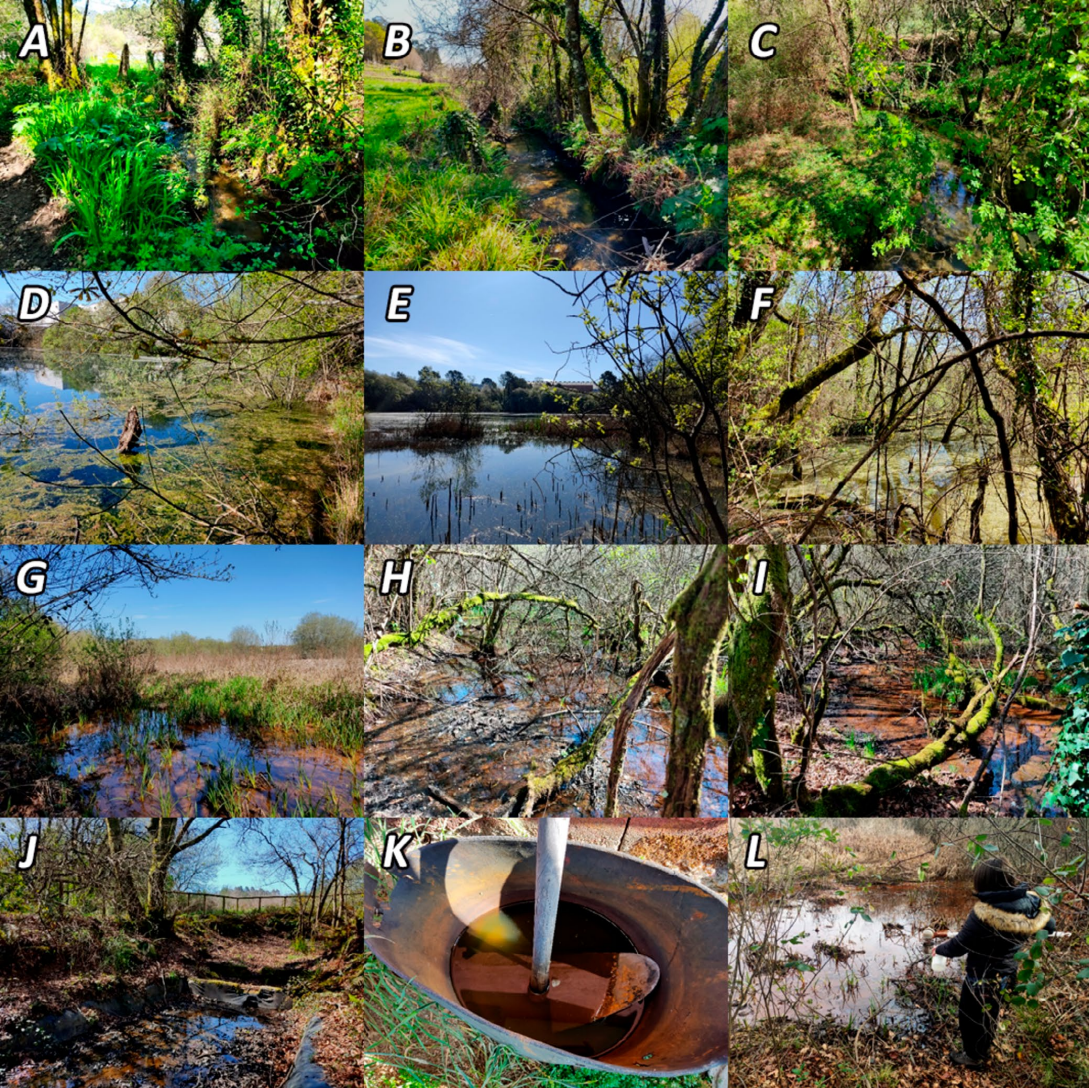
Photographs showing the sampling points during spring season (A: River Folón, B: River Penedo, C: River San Simón, D: Lagoon Sisargas, E: Lagoon Granxa, F: Lagoon Orbenlle, G: Swamp North, H: Swamp East, I: Swamp West, J: Pond Granxa, K: Container Granxa) and the dipping technique for mosquito larvae capture (L).

The field research was conducted in 11 sampling points selected according to their accessibility and representativeness of the diversity of aquatic ecosystems of the wetland by in situ observation and the use of geographic information systems (GIS) such as Google Earth and Quantum GIS (3.8 QGIS version) (QGIS Development Team 2009) (Figure 1). The sampling points ranged from altitudes of 15 to 66 masl and were classified according to hydroregime (temporary, when desiccation occurs in dry season; or permanent, when remains with a layer of water throughout the year) and water body type (river, lagoon, swamp, pond and artificial container) (Table 1). In general, the study area vegetation is characterized by the presence of riparian forests ( *Alnus glutinosa* , Betulaceae; *Fraxinus excelsior* , Olaceae; *Laurus nobilis* , Lauraceae; and *Salix atrocinerea* , Salicaceae) and small oak woods ( *Quercus robur* , Fagaceae) surrounded by mixed forests of *Pinus pinaster* (Pinaceae) and *Eucalyptus globulus* (Myrtaceae) (Requejo and Castro 2017) (Figure 2). Although all the aquatic ecosystems under study are affected by the activity of the industrial state (including manufacturing of automotive and industrial components, granite quarrying, food and pharmaceutical production, etc.), the swamps show the greatest signs of contamination due to polluted discharges.

**TABLE 1:** Information of sampling points including code, water body type and name, hydroregime, altitude in meters (Alt), total number of samples (*n*), number of samplings with mosquitoes (*n*+) and relative frequency of mosquitoes (*F*%) [(*n*+/*n*)*100].

Code	Water body name	Hydroregime	Alt	*n*	*n*+	*F*%
RF	River Folón	Permanent	66	23	0	0
RP	River Penedo	Permanent	35	23	1	4.35
RS	River San Simón	Permanent	17	23	2	8.7
LS	Lagoon Sisargas	Permanent	15	23	3	13
LG	Lagoon Granxa	Permanent	18	23	5	21.74
LO	Lagoon Orbenlle	Permanent	21	23	13	56.52
SN	Swamp North	Temporary	21	19	13	68.42
SE	Swamp East	Temporary	15	18	14	77.78
SW	Swamp West	Temporary	17	19	16	84.21
PG	Pond Granxa	Temporary	34	9	8	88.89
CG	Container Granxa	Temporary	15	15	11	73.33

### 2.2 Mosquito Sampling and Processing

Larval sampling is necessary to identify mosquito proliferation hotspots and prevent nuisance and epidemiological risks associated with adult activity (Russell 1999). Since most mosquito species complete their aquatic development within an average of 15 days (Beaty and Marquardt 1996), sampling was performed fortnightly between January and December 2022. The standardized dipping technique for mosquito capture (Service 1993) was used by introducing a 500 mL dipper in those areas of the water body most suitable for harboring mosquitoes (edges, shallow spots and areas surrounded with aquatic vegetation) within a radius of 1–5 m (Figure 1). Ten dips were taken at each sampling point whenever the volume of water was enough. Mosquito larvae and pupae were transported to the laboratory and reared at room temperature in plastic jars with water from their breeding grounds until reaching the IV instar larvae and adult stage, respectively. Those specimens that did not survive were removed and identified for inclusion in the final database. All specimens were fixed in 70% ethanol and identified to species under a binocular magnifier and an optical microscope according to the morphological criteria of Becker et al. (2020). Samples remain stored in the scientific collection of the Aquatic Entomology Laboratory in the Faculty of Biology at Universidade de Vigo (Vigo, Galicia, Spain).

### 2.3 Climatic Variables

Different climatic variables that characterized the study area during the sampling period (January to December 2022) were considered (maximum, minimum and mean temperature, mean relative humidity, accumulated precipitation and mean wind speed). These data were extracted from the closest weather station to the study area (less than 4 km away, in Atios, O Porriño) (MeteoGalicia 2023). The winter months (January to March) were the coldest, recording an average of 9.3°C and minima below 0°C (−3.7°C) with occasional maxima above 20°C; as well as relatively regular (34% of rainy days) and moderate rainfall (226 L/m^2^). The spring months (April to June) had generally warm temperatures (mean of 16°C) with maxima of almost 34°C and occasional minima below 0°C; and a rainfall dynamic similar to the previous period (38% of rainy days with 258 L/m^2^). The summer months (July to September) were the warmest, with a mean temperature of 21.3°C, occasional maxima close to 40°C and minima of 6°C; while rainfall drastically decreased in frequency (13% of rainy days) and amount (114 L/m^2^). The autumn months (October to December) recorded more spring‐like temperatures, with an average of 14°C, maxima of 29°C and minima of −1.2°C; being a particularly rainy period (67% of rainy days with 1309 L/m^2^). Mean relative humidity was high in all seasons (> 83%), with the highest values in winter and autumn (90%). Mean wind speed was similar throughout the study period, being lower in winter (3.6 km/h) and around 4.8 km/h the rest of the year.

### 2.4 Data Analysis

Statistical analyses were performed using the BiodiversityR, car, lme4, MASS, Matrix, MuMIn, pgirmess, and vegan packages of version 4.2.0 of the R software (R Core Team 2022).

#### 2.4.1 Functional Groups

Following the recommendations of Schäfer and Lundström (2001), mosquito species were classified into functional groups according to their ecological characteristics (oviposition sites, overwintering stage, preferred host and number of generations) in order to facilitate the identification of nuisance species and the comprehension of the results by the general public and the administration. More specifically, the classification adapted to the mosquitoes of Spain by Bueno‐Marí and Jiménez‐Peydró (2011) was applied.

#### 2.4.2 Diversity Indices Comparison

Frequency, abundance, species richness (S), Shannon–Wiener's (H^0^) and Simpson's (DS) diversity indices were calculated for each group of environmental variables (hydroregime, water body type and season). Since the data did not follow a normal distribution according to the Shapiro–Wilk test (*p* < 0.05), the Kruskal–Wallis test was applied to determine differences between groups at a significance level of 0.05 (Kruskal and Wallis 1952). Subsequently, a Tukey's range test (post hoc test) was applied to identify which groups were significantly different from others.

#### 2.4.3 Generalized Linear Mixed Models (GLMM)

Two generalized linear mixed models were performed to determine the environmental variables (explanatory variables) affecting the abundance and richness of mosquitoes (response variables) in the study area, respectively. The environmental data included qualitative (hydroregime, water body type and season) and quantitative variables such as maximum, minimum and mean temperature (°C), mean relative humidity (%), accumulated precipitation (L/m^2^) and mean wind speed (km/h) corresponding to the 15 and 7 days prior to the sampling date. Considering the high over‐dispersion in the abundance data (variance larger than mean), a negative binomial generalized linear mixed model (NBGLMM) (logit link function) was chosen to analyze this variable. A GLMM with a Poisson distribution (logit link function) was applied to determine the richness as the best fitting model for a low over‐dispersion data. In both models, the environmental variables were analyzed as fixed effects while the sampling point was included as a random effect. A manual forward‐stepwise approach was employed for model selection considering the results of the ANOVA test for model comparison (*p* < 0.05). The final models were those with the lowest Akaike Information Criterion (AIC) and highest coefficients of determination (*R* ^2^), both marginal (proportion of variance explained by the fixed effects) (*R* ^2^m) and conditional (proportion of variance explained by the fixed and random effects) (*R* ^2^c). No multicollinearity problems were detected in any of the final models since all the variables showed a low variance inflation factor (VIF < 2).

## 3 Results

### 3.1 Abundance and Distribution of Mosquito Species and Functional Groups

**FIGURE 3:**
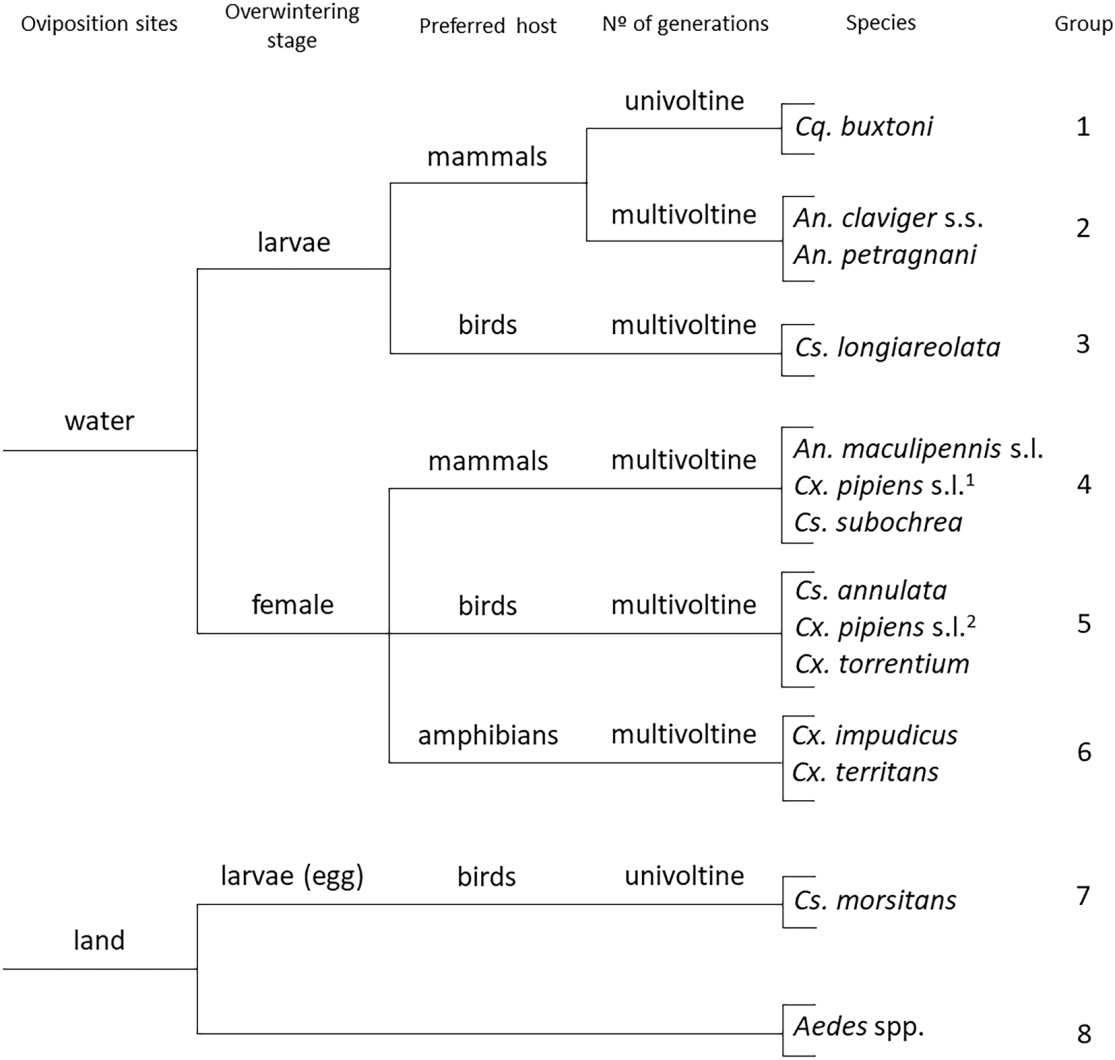
Functional group classification of the mosquito species identified in the wetland of Gándaras de Budiño (Galicia, NW Spain). ^1^ *Culex pipiens* biotype *molestus*, ^2^ *Culex pipiens* biotype *pipiens*.

A total of 766 mosquitoes belonging to 11 species of the genus *Culex* (57%), *Culiseta* (30%) and *Anopheles* (13%) were identified. The most abundant species was by far *Culex pipiens* s.l. Linnaeus, 1758 (*N* = 287, 37.5%), followed by *Culiseta annulata* (Schrank, 1776) (*N* = 133, 17.4%), *Anopheles maculipennis* s.l. Meigen, 1818 (*N* = 98, 12.8%), *Culex territans* Walker, 1856 (*N* = 87, 11.4%) and *Culiseta longiareolata* Macquart, 1838 (*N* = 71, 9.3%); whereas *Culex torrentium* Martini, 1925 (*N* = 34, 4.4%), *Culex impudicus* Ficalbi, 1890 (*N* = 26, 3.4%), *Culiseta subochrea* (Edwards, 1921) (*N* = 19, 2.5%), *Culiseta morsitans* Theobald, 1901 (*N* = 9, 1.2%), *Anopheles claviger* s.s. (Meigen, 1804) (*N* = 1, 0.1%) and *Anopheles petragnani Del Vecchio, 1939* (*N* = 1, 0.1%) occurred in smaller numbers. In addition, five adults of *Coquillettidia buxtoni* (Edwards, 1923) and a specimen of *Aedes* spp. were observed and hand collected when they approached the researchers (the latter with biting intention) during the June samplings in the swamp area. A total of eight functional groups of mosquitoes were identified in the wetland (Figure 3).

**FIGURE 4:**
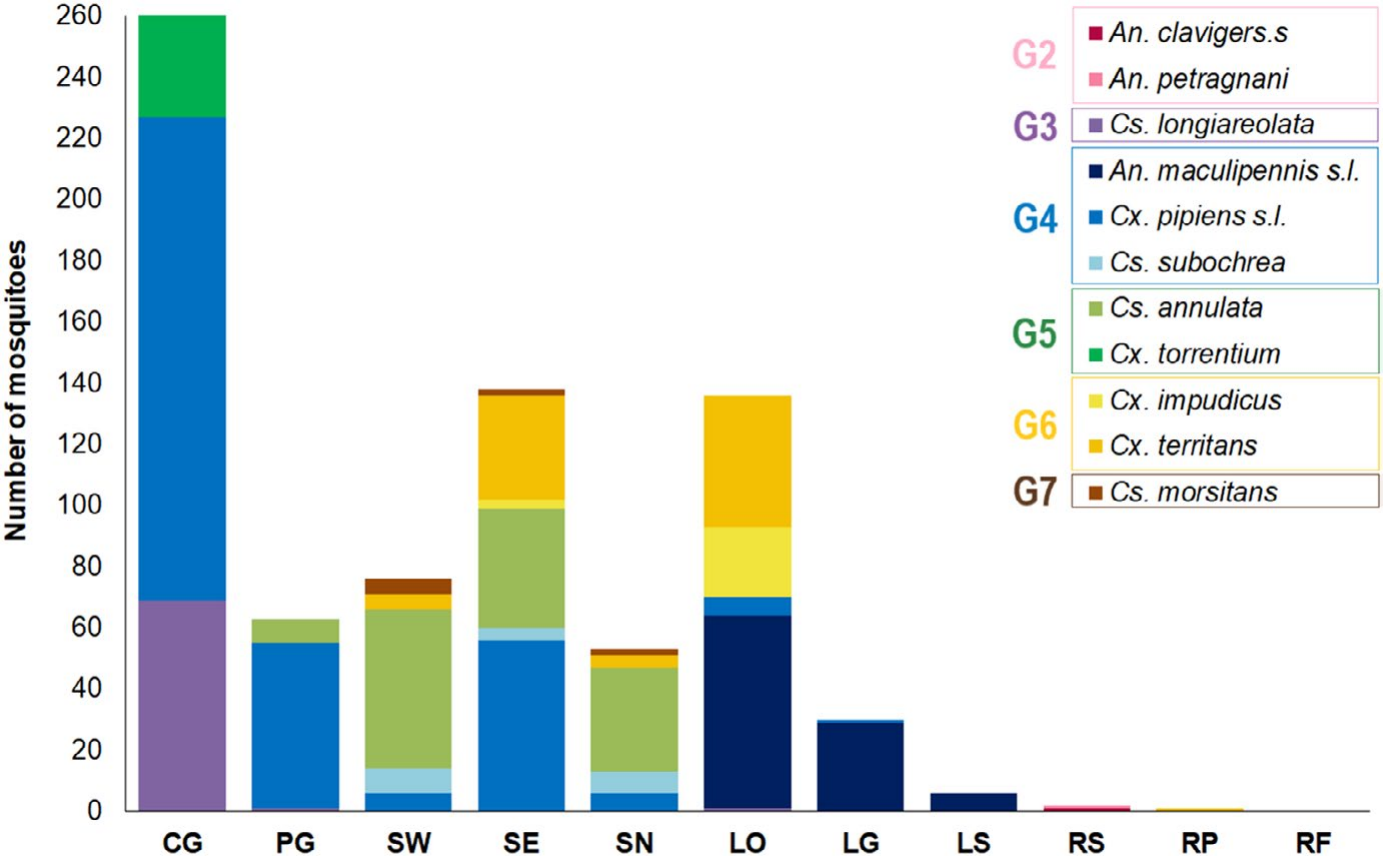
Total number of mosquitoes captured at each sampling point regarding species and functional groups (G2–G7).

The presence of mosquitoes was confirmed in practically all the sampling points (91%) with a different frequency, abundance, richness and proportion of species (Table 1, Figure 4). *Culex pipiens* s.l. was the best distributed species (64%), followed by *Cs. annulata* and *Cx. territans* (45%), *Cs. longiareolata* (27%) and *Cx. impudicus* (18%); which were found breeding in different types of water bodies (Figure 4). *Anopheles maculipennis* s.l. was exclusively observed in lagoons, *Cs. subochrea* and *Cs. morsitans* in swamps (27%), and *An. claviger* s.s and *An. petragnani* only in a river (9%) (Figure 4). Functional groups 4 and 5 were dominants in the study area (Figure 4).

### 3.2 Seasonal Dynamics and Larval Habitats of Mosquito Species

**FIGURE 5:**
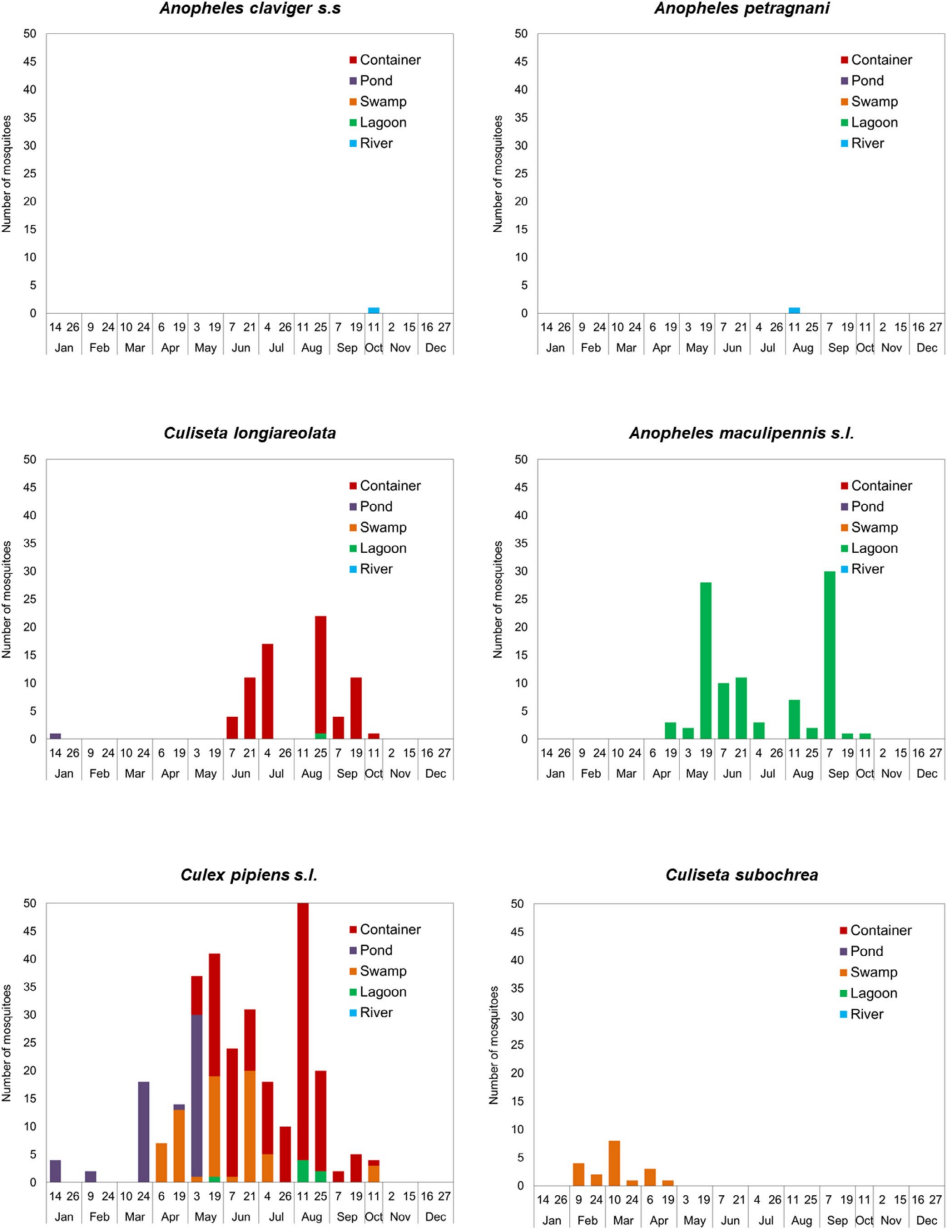
Total number of specimens captured during the study period (January–December 2022) in relation to larval habitats for each of the mosquito species included in functional groups 2–4.

**FIGURE 6:**
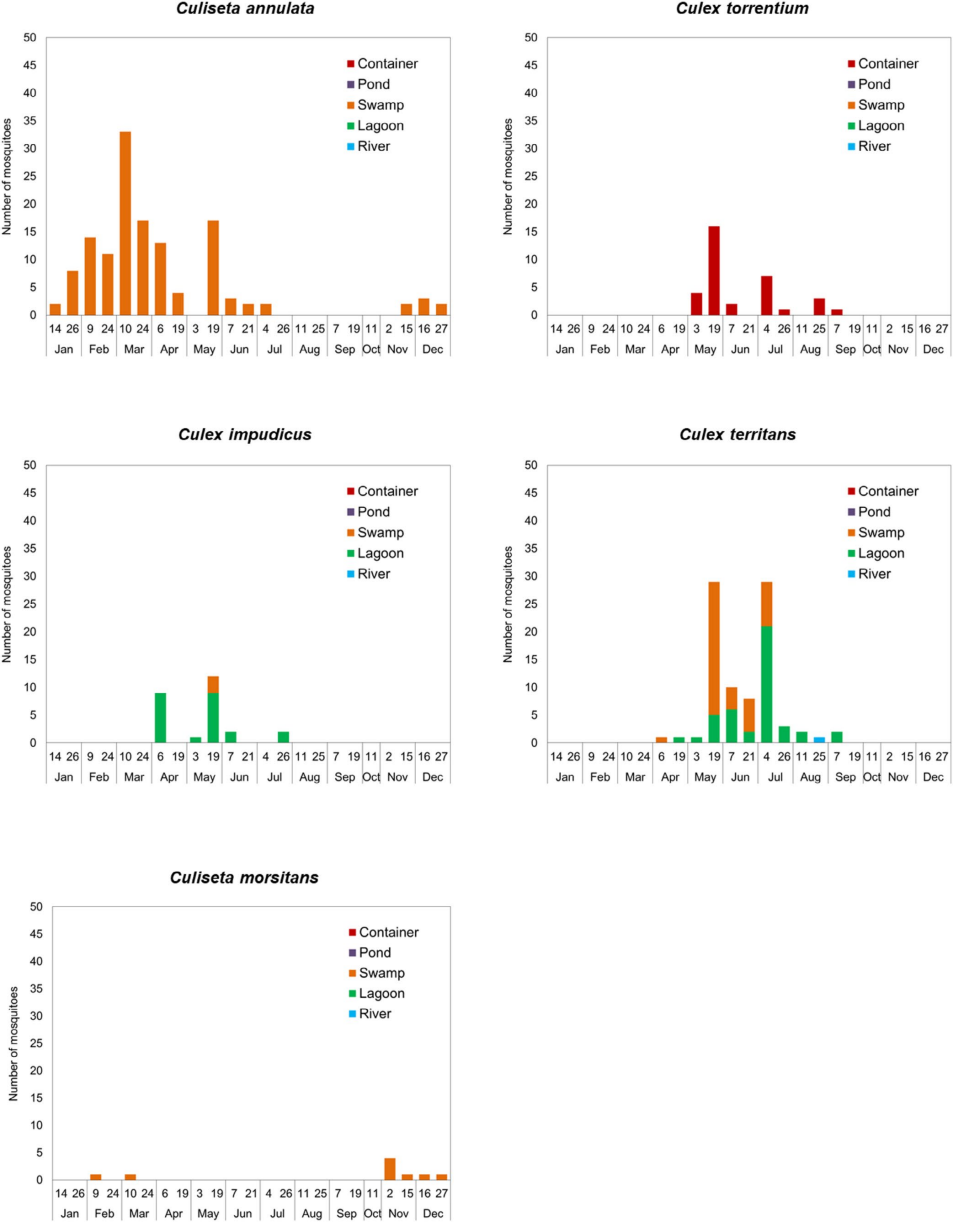
Total number of specimens captured during the study period (January–December 2022) in relation to larval habitats for each of the mosquito species included in functional groups 5–7.

Mosquito species showed different seasonal dynamics and larval habitats in the study area (Figures 5 and 6). *Anopheles petragnani* and *An. claviger* s.s. were observed in a river in August and October, respectively (Figure 5). *Culiseta longiareolata* was found breeding preferably in the artificial container (occasionally in the pond and lagoons) in the spring and summer seasons, peaking in July and August (Figure 5). *Anopheles maculipennis* s.l. larvae were found exclusively in lagoons between April and October, with two activity peaks at the end of May and the beginning of September (Figure 5). *Culex pipiens* s.l. was the only species occurring in all seasons and almost in all types of water bodies (excluding rivers), being found breeding from January to October with several peaks of abundance that reached a maximum in early August (Figure 5). *Culiseta subochrea* was detected only in swamps from February to April (Figure 5).

*Culiseta annulata* displayed a preference for breeding in swamps (occasionally the pond), where it was the most abundant and longest‐active species (only absent between August and October, with peaks of abundance in March and May) (Figure 6). *Culex torrentium* was only detected breeding in the artificial container with intermittent activity from May to September (Figure 6). *Culex impudicus* larvae were found in lagoons and swamps with occasional occurrences in April, May, and July; while *Cx. territans* was observed mainly in lagoons and swamps (exceptionally rivers) from April to September, reaching the highest larval abundance in May and July (Figure 6). *Culiseta morsitans* larvae were observed exclusively in swamps and in small numbers from February to March and from November to December (Figure 6).

### 3.3 Mosquito Diversity According to Habitat Characteristics

Statistically significant differences in abundance and diversity indices were observed between groups of the three habitat characteristics (hydroregime, water body type and season) regarding median values (*p* < 0.05) (Table 2). Both abundance and species richness were significantly higher in temporary water bodies (artificial container, pond and swamps). Although the Shannon's diversity index was very low in all cases (H^0^ < 1), temporary waters such as the artificial container recorded significantly higher values. Simpson's dominance index revealed significantly high dominance of species in permanent water bodies (rivers and lagoons) (SD = 1). Regarding the seasons, spring was the one with significantly higher values of abundance and species richness, as well as a lower Simpson's dominance index.

**TABLE 2:** Sampling points information and mosquito frequency, abundance, and diversity regarding habitat characteristics.

Habitat characteristics		*n*	*n*+	*F*%	N		S		H^0^		DS	
					Me	K–W	Me	K–W	Me	K–W	Me	K–W
Hydroregime	Permanent	195	66	33.85	0^a^	*H*: 24.58 df: 1 *p*: < 0.001	0^a^	H: 19.02 df: 1 *p*: < 0.001	0^a^	H: 17.05 df: 1 *p*: < 0.001	1^a^	H: 12.04 df: 1 *p*: < 0.001
	Temporary	24	19	79.16	7^b^		2^b^		0.15^b^		0.47^b^	
Water body	River	69	3	4.35	0^a^	H: 76.07 df: 4 *p*: < 0.001	0^a^	H: 72.58 df: 4 *p*: < 0.001	0^a^	H: 45.90 df: 4 *p*: < 0.001	1^a^	H: 66.99 df: 4 *p*: < 0.001
	Lagoon	69	21	30.44	0^a^		0^a^		0^a^		1^a^	
	Swamp	56	42	75	2^b^		1^b^		0^a^		0.44^b^	
	Pond	9	8	88.88	2^b^		1^b^		0^a^		0^b^	
	Container	15	11	73.33	11^b^		2^b^		0.64^b^		0.5^b^	
Season	Winter	60	19	31.67	0^a^	H: 15.40 df: 3 *p*: 0.001	0^a^	H: 13.15 df: 3 *p*: 0.004	0^a^	H: 10.24 df: 3 *p*: 0.02	1^a^	H: 10.37 df: 3 *p*: 0.02
	Spring	70	38	54.28	1^b^		1^b^		0^a^		0.6^b^	
	Summer	48	17	35.42	0^a^		0^a^		0^a^		1^a^	
	Autumn	40	11	27.5	0^a^		0^a^		0^a^		1^a^	

*Note:* Includes total number of samples (*n*), number of samples with mosquitoes (*n*+) and relative frequency of mosquitoes (*F*%) [(*n*+/*n*)*100], as well as median values (Me) and Kruskal–Wallis test results (K–W) (H: test statistics, df: degree of freedom, *p*: *p* value) for mosquito abundance (N), species richness (S), Shannon–Wiener's (H^0^) and Simpson's (DS) diversity indices. Different superscript letters in median values indicate statistically significant differences between groups according to the Tukey's range test.

### 3.4 Climatic Variables Determining Larval Abundance and Species Richness of Mosquitoes

The best‐fitting model of larval abundance of mosquitoes explains 78.16% of the variance data (Table 3), whereas the best‐fitting model of species richness of mosquitoes explains the 70.95% (Table 4). Both biological variables are influenced in a similar way by the same environmental variables as they increase with maximum ambient temperature and decrease with the mean relative humidity of the 15 days prior to sampling date; being also favored by temporary water bodies (pond and artificial container) compared to permanent ones (rivers and lagoons) (Tables 3 and 4).

**TABLE 3:** Negative binomial generalized linear mixed model (NBGLMM) for mosquito larval abundance.

Explanatory variables	NBGLM statistics					
	*β* ± SE	*Z*	*p*	AIC	*R* ^2^m (%)	*R* ^2^c (%)
Intercept	9.89 ± 2.95	3.35	< 0.001			
*T*max15 (°C)	0.11 ± 0.03	3.63	< 0.001			
Hum15 (%)	−0.12 ± 0.03	−4.41	< 0.001			
Waterbody [container]	Ref.					
Waterbody [pond]	0.39 ± 1.05	0.38	0.71			
Waterbody [lagoon]	−2.20 ± 0.80	−2.77	0.006			
Waterbody [swamp]	−0.29 ± 0.82	−0.36	0.72			
Waterbody [river]	−6.04 ± 1.00	−6.02	< 0.001			
Global model				670.9	74.71	78.16

*Note:* Global model: Mosquito larval abundance = *T*max15 + Hum15 + Waterbody + (1|Samplingpoint).

Abbreviations: AIC, Akaike information criterion; *F*, *F* statistics; Hum15, mean relative humidity 15 days prior to sampling; *p*, *p* value; *R* ^2^c, coefficient of determination (conditional); *R* ^2^m, coefficient of determination (marginal); Ref., reference category; SE, standard error; *T*max15, maximum temperature 15 days prior to sampling; *Z*, *Z* value; *β*, parameter estimates.

**TABLE 4:** Generalized linear mixed model with a Poisson distribution (GLMM) for mosquito species richness.

Explanatory variables	NBGLM statistics					
	*β* ± SE	*Z*	*p*	AIC	*R* ^2^m (%)	*R* ^2^c (%)
Intercept	4.18 ± 1.78	2.35	0.019			
*T*max15 (°C)	0.04 ± 0.02	2.57	0.010			
Hum15 (%)	−0.06 ± 0.02	−3.50	< 0.001			
Waterbody [container]	Ref.					
Waterbody [pond]	0.001 ± 0.73	0.002	0.998			
Waterbody [lagoon]	−1.24 ± 0.57	−2.17	0.030			
Waterbody [swamp]	−0.01 ± 0.55	−0.01	0.991			
Waterbody [river]	−3.60 ± 0.79	−4.57	< 0.001			
Global model				367.8	65.96	70.95

*Note:* Global model: Mosquito species richness = *T*max15 + Hum15 + Waterbody + (1|Samplingpoint).

Abbreviations: AIC, Akaike information criterion; *F*, *F* statistics; Hum15, mean relative humidity 15 days prior to sampling; *p*, *p* value; *R* ^2^c, coefficient of determination (conditional); *R* ^2^m, coefficient of determination (marginal); Ref., reference category; SE, standard error; *T*max15, maximum temperature 15 days prior to sampling; *Z*, *Z* value; *β*, parameter estimates.

## 4 Discussion

The species richness found in the Gándaras de Budiño is relatively high considering the results obtained in other similar studies carried out in the Iberian Peninsula (Bueno‐Marí et al. 2008; González et al. 2021; Martínez‐Barciela et al. 2023). The species composition observed in the study area is very similar to other regions of northern Spain, where *Culex* mosquitoes are predominant and the presence of *Aedes* is reduced (González et al. 2021; Martínez‐Barciela et al. 2023). The previous knowledge on the mosquito populations of the Gándaras de Budiño (Martínez‐Barciela et al. 2021, 2023) has been updated, confirming the presence of the six species already recorded (*An. maculipennis* s.l., *Cx. pipiens* s.l., *Cx. territans*, *Cs. annulata*, *Cs. morsitans*, and *Cq. buxtoni*) and reporting for the first time another six (*An. claviger* s.s, *An. petragnani*, *Cx. impudicus*, *Cx. torrentium*, *Cs. longiareolata*, and *Cs. subochrea*), along with an unidentifiable specimen of *Aedes*. The classification of these species into functional groups makes it possible to distinguish eight groups with different larval habitats, seasonal dynamics, and health implications.

Group 1 consists only of *Cq. buxtonii*, which was punctually detected in its adult stage in June. The dipping technique was not adequate for the particular capture of this species, being necessary an intensive sampling with an entomological net (Martínez‐Barciela et al. 2021) to unanchor these larvae from the aquatic plants to which they attach to obtain oxygen (Becker et al. 2020). Despite being considered a univoltine species (Bueno‐Marí and Jiménez‐Peydró 2011), previous studies carried out in the wetland revealed the presence of larvae at different months of the year that are incompatible with overwintering (Martínez‐Barciela et al. 2021), suggesting that it may behave as a multivoltine species in southern Europe. Females could be very numerous and aggressively bite humans and cattle in open areas (Gutsevich et al. 1974), but no nuisance was reported in the wetland. There is also no evidence that this species plays a role as a disease vector, so its presence in the wetland does not pose a health risk.

Group 2 is composed by two morphologically and ecologically very close species with a marked host preference for large domestic animals (Becker et al. 2020): *An. petragnani* and *An. claviger* s.s. *Anopheles petragnani* plays no role in pathogens transmission and, even though *An. claviger* s.s. is a potential vector of malaria, does not pose an epidemiological risk due to its small populations (Becker et al. 2020). Both were detected only once in the same river, in summer and autumn, respectively. This coincides with their known seasonal dynamics and breeding preferences for water bodies of fluvial origin and shaded situations (Becker et al. 2020; Martínez‐Barciela, Polina, and Garrido 2024).

Group 3 corresponds to *Cs. longiareolata*, a species capable of breeding in a great diversity of aquatic environments (Becker et al. 2020), being found in artificial containers, ponds and lagoons. Adults can be found from February to November in temperate climatic zones (Becker et al. 2020), while larvae were found overwintering in January and being active from June to October. Females rarely bite humans outside, showing preference for birds to which can transmit avian malaria (Seidel et al. 2013).

Group 4 includes *An. maculipennis* s.l., *Cx. pipiens* s.l. biotype *molestus*, and *Cs. subochrea*. The former is a complex of sibling species that differ in their biology and behavior, being represented in Spain primarily by *An. atroparvus* and secondarily by *An. maculipennis* s.s. (Taheri et al. 2024). The larvae were found exclusively in lagoons, exposed to the sun and in presence of green algae, coinciding with the documented breeding preferences of *An. atroparvus* (Becker et al. 2020). Larval activity lasted from April to October, suggesting that females overwinter from November to February, similar to what has already been observed in other regions of southern Europe (Becker et al. 2020). Females are strongly mammophilic, feeding mainly on cattle and occasionally on humans, both outdoors and indoors (Šebesta et al. 2011; de la Martinez‐Puente et al. 2013; Becker et al. 2020). Although *An. maculipennis* s.s. is considered a vector of minor importance in malaria transmission, *An. atroparvus* is the main malaria vector in Europe (Piperaki and Daikos 2016). However, the absence of endemic circulation of the parasite (*Plasmodium* spp.) in Spain and the refractoriness of this mosquito to transmit *P. falciparum* tropical strains (responsible for the majority of imported classes), makes the epidemiological risk associated with *An. maculipennis* s.l. low (Taheri et al. 2024). *Culex pipiens* s.l. is an assemblage of morphologically similar species (Pipiens Assemblage) that in Europe includes *Cx. pipiens*, widespread in the Holartic region, and *Cx. quinquefasciatus*, present in the tropics and subtropics (Harbach 2012). *Culex pipiens* biotype *molestus* prefer to bite humans while biotype *pipiens* mainly feed on birds, (Osorio et al. 2014). Larvae are able to inhabit nearly every kind of water collection (Becker et al. 2020) as evidenced by its presence in almost all the sampling points, registering higher abundances in the artificial container. Larvae were observed from January until October as this species can develop up to several generations per year, overwintering in adult stage in the coldest months (Becker et al. 2020). *Culex pipiens* s.l. plays an increasing role in the transmission and enzootic circulation of WNV in Europe (Fros et al. 2015) and it is the principal bridge vector of the disease worldwide (Becker et al. 2020), being also a competent vector for Sindbis virus (SINV) (Jansen et al. 2023) and Usutu virus (USUV) (Krambrich et al. 2024). On the contrary, *Cs. subochrea* is not associated with disease transmission even though it can bite both domestic animals and humans (Becker et al. 2020). This species can present several generations per year (Becker et al. 2020), but larvae were only found from February to April breeding in the swamps.

Group 5 consists of *Cs. annulata*, *Cx. pipiens* s.l. biotype *pipiens*, and *Cx. torrentium*. These are ornithophilic species that can also bite mammals (including humans) (Osorio et al. 2014; Becker et al. 2020), being potential bridge vectors for different viruses (Becker et al. 2020). While *Cs. annulata* can transmit Tahyna virus (Ribeiro et al. 1988), *Cx. torrentium* is a competent vector for Sindbis and West Nile viruses (Hesson et al. 2015; Jansen et al. 2019, 2023). Although it is known that *Cs. annulata* can be a cause of nuisance even in low abundances (Medlock and Leach 2012), no bites were reported during the samplings. This species may overwinter as an adult in Central Europe (Becker et al. 2020), but larvae were observed in the wetlands since January, confirming the existence of larval overwintering episodes in southern Europe (Bueno‐Marí and Jiménez‐Peydró 2011). The lower abundance in summer and autumn is related to the drying of their larval biotopes: the swamps and the pond. In contrast, *Cx. torrentium* appears only in the warmer months (Becker et al. 2020), from May to September, breeding exclusively in the artificial container.

Group 6 comprises two species with no health interest that mainly feeds on amphibians: *Cx. impudicus* and *Cx. territans* (Becker et al. 2020). Both breed in stagnant and preferably shaded freshwaters, being found in the swamps and a lagoon with high vegetation cover. Although *Cx. territans* rarely breeds in highly polluted waters (Becker et al. 2020), the highest larval abundances were recorded precisely in the swamps. As observed in the wetland, larvae appear from early spring to late summer (Becker et al. 2020).

Group 7 only includes *Cs. morsitans*, an ornithophilic species that rarely attacks human and may be carrier of West Nile (McIntosh et al. 1967) and Sindbis viruses (Bergqvist et al. 2015). Its presence in the wetland is noteworthy as it is the only place in Galicia where this species has been found so far (Martínez‐Barciela et al. 2020; Martínez‐Barciela et al. 2023; Martínez‐Barciela, Polina, and Garrido 2024). Matching prior knowledge (Becker et al. 2020), larvae were found between autumn and winter, breeding preferably in swamps.

Group 8 would contain at least one *Aedes* species of diurnal activity and attraction to humans. However, its epidemiological interest cannot be defined as the species could not be identified. Its isolated observation in the study area suggests that it may occur in low population densities, but the nonapplication of other methodologies such as ovitraps and traps baited with CO_2_ could have underestimated its presence in the wetland (ECDC 2012, 2014).

Almost all functional groups contain some mosquito capable of transmitting diseases. However, either because of their ecology, small populations or the absence of endemic circulation of the pathogens, most of the species do not currently pose an epidemiological risk in the wetland. Only *Cx. pipiens* s.l. (Group 4) and *Cx. torrentium* (Group 5) represent an emerging risk given their high competence to transmit WNV (Fros et al. 2015; Jansen et al. 2019, 2023), already endemic in southern Spain (Magallanes et al. 2024). The regular presence in the wetland of birds with a potential role in the WNV circulation such as the common blackbird ( *Turdus merula* Linnaeus, 1758), Eurasian magpie ( *Pica pica* (Linnaeus, 1758)), house sparrow ( *Passer domesticus* (Linnaeus, 1758)) and common coot ( *Fulica atra* Linnaeus, 1758) (Rizzoli et al. 2015; Magallanes et al. 2024; Naturaspain 2024), together with the high availability of human hosts linked to the activity of the industrial state; make this wetland an area of great epidemiological interest.

Risk assessment is not only affected by the mosquito species present and their access to pathogens and humans, but also by environmental conditions and population density (Service 1993). Although the abundance of immatures may not necessarily translate into the same number of adults, high larval densities allow one to identify the areas of greatest mosquito proliferation (Russell 1999). Generally, the characteristics of larval habitats will influence the diversity and abundance of mosquitoes (Russell 1999). Although a greater total number of species were observed colonizing water bodies rich in aquatic vegetation and organic matter such as swamps and lagoons, both the frequency and the median values of abundance and diversity were significantly higher in temporal environments such as the artificial container, the pond, and the swamps. Permanently flooded and deep‐water habitats with an established and diverse fauna may harbor a greater variety of mosquito species, but usually produce lower abundance (Russell 1993). As observed in the study area (especially in swamps), organically polluted waters favor mosquito proliferation by providing more nutrients (Carlson and Knight 1987) and by decreasing the survival and activity of predators (Mian et al. 1986; Russell 1999). In addition to being influenced by larval habitat characteristics, mosquito abundance and diversity are also determined by climate (Roiz et al. 2014). Larval abundance and species richness exhibit a similar pattern in the study area as both register significantly higher values in spring, increasing with maximum ambient temperatures and decreasing with mean relative humidity of the 15 days prior to sampling. Considering that this is the average time required for mosquitoes to complete their aquatic cycle (Beaty and Marquardt 1996), these environmental variables would not only affect larval development but also the oviposition activity of females. It is widely documented that ambient temperatures (while not exceeding 40°C) are positively related to mosquito abundance by favoring their survival and larval productivity (Lafferty 2009; Tabachnick 2010; Roiz et al. 2014). The few studies on the relationship between humidity and mosquitoes indicate a generally positive effect on their activity and egg development up to 90% relative humidity (Brown et al. 2023). However, in the present study area, this value is frequently reached, resulting in a slightly negative effect on larval abundance and species richness. High humidity may cause too little surface tension in larval habitats, negatively affecting the ability of larvae to access atmospheric oxygen and nutrients (Singh and Micks 1957; Brown et al. 2023).

The eradication of mosquitoes is not practicable, but reduction through a management approach is usually feasible (Russell 1999). Mosquito control is most effectively done at the larval stages as the individuals are spatially concentrated (Dale and Knight 2008). The Integrated Mosquito Management (IMM) approach recommends decreasing their breeding habitats, promoting native fauna, and educating the public (Martinou et al. 2020; Dwork et al. 2022). Temporary and highly polluted water bodies have been identified as the main breeding sites for the species of major epidemiological interest in the study area. Avoiding drainage (Chase and Knight 2003; Medlock and Vaux 2015) and adequately treating contaminated waters of the wetland could substantially reduce *Cx. pipiens* s.l. populations by decreasing available food for larvae (Mian et al. 1986), favoring the survival of their natural predators (larvivorous fishes, copepods, dragonfly nymphs, etc.) (Mian et al. 1986; Dale and Knight 2008) and increasing the diversity of other aquatic invertebrates with which they compete for resources (Elono et al. 2010; Dwork et al. 2022). Complementarily, the administration should encourage and support the elimination of artificial containers both on public ways and private properties through education campaigns aimed at the general public and companies. The elimination of these potential breeding sites is especially relevant not only to reduce the populations of *Cx. pipiens* s.l. and *Cx. torrentium*, but also to prevent the proliferation of *Aedes albopictus* (Skuse, 1894), an invasive mosquito capable of transmitting dengue, Zika, and chikungunya, which has already been detected in areas near the wetland (Martínez‐Barciela, Polina, Pereira, et al. 2024). The application of these measures is especially relevant in spring and summer as larval densities are expected to be higher. The use of other control methods should be considered only under a serious mosquito nuisance and high epidemiological risk, and always considering the results of vector surveillance and expert guidance.

## 5 Conclusions

The Gándaras de Budiño wetland contains different types of water bodies that support a great species richness of mosquitoes. Although many of them are potential vectors, only two are currently relevant in the WNV transmission: *Cx. pipiens* and *Cx. torrentium*. This, added to the presence in the wetland of certain birds with a potentially important role in the circulation of the virus and the wide availability of human hosts linked to the activity of the industrial estate, makes this a place of special epidemiological interest. The characterization of the larval habitats and the seasonal dynamics of the mosquitoes has made it possible to identify the artificial containers and polluted waters as the main breeding sites of the species of major sanitary interest, as well as late spring and summer as their period of major activity. Although the risk of autochthonous transmission is currently low due to the nonendemism of WNV in northwestern Spain, it is crucial to keep an active and integrated vector surveillance (applying different and complementary sampling methods) in order to implement the most efficient and environmentally responsible prevention and control measures. The presence of mosquitoes in a wetland should not go against the conservation interest of these valuable spaces, but must be another reason for their care and protection since a healthy and equilibrated ecosystem is less vulnerable to becoming a health concern.

## Author Contributions

**Yasmina Martínez‐Barciela:** conceptualization (lead), data curation (equal), formal analysis (lead), funding acquisition (supporting), investigation (equal), methodology (equal), writing – original draft (lead), writing – review and editing (equal). **Alejandro Polina:** conceptualization (supporting), data curation (equal), investigation (equal), methodology (equal), writing – original draft (supporting), writing – review and editing (equal). **Josefina Garrido:** conceptualization (supporting), data curation (supporting), funding acquisition (lead), investigation (supporting), project administration (lead), supervision (lead), writing – original draft (supporting), writing – review and editing (equal).

## Conflicts of Interest

The authors declare no conflicts of interest.

## Supporting information


Appendix S1

## Data Availability

The required data are uploaded as Supporting Information.
